# Mode of Delivery Does Not Influence Postpartum Hypercoagulability Measured by Thrombin Generation or Thromboelastometry

**DOI:** 10.1055/s-0039-3402807

**Published:** 2020-01-07

**Authors:** Boriana Guimicheva, Lara N. Roberts, Jignesh P. Patel, Devi Subramanian, Roopen Arya

**Affiliations:** 1 Department of Haematological Medicine, King's Thrombosis Centre, King's College Hospital NHS Foundation Trust, London, United Kingdom; 2 Women's Health, King's College Hospital NHS Foundation Trust, London, United Kingdom; 3 Institute of Pharmaceutical Science, King's College London, London, United Kingdom

**Keywords:** enoxaparin, vaginal delivery, caesarean section, thrombin generation, thromboelastometry

## Abstract

**Introduction** Venous thromboembolism (VTE) is a significant cause of maternal mortality with the greatest risk postpartum. Mode of delivery influences VTE risk, with emergency caesarean section (CS) associated with the highest risk (CS). Thromboprophylaxis is recommended for selected women to reduce the risk of VTE.

We aimed to investigate the impact of mode of delivery and thromboprophylaxis on hypercoagulability as measured by thromboelastometry (TEM) and thrombin generation (TG) in women at high VTE risk.

**Materials and Methods** Blood was collected from 99 pregnant women with VTE risk factors at up to five time points from pre- (T1) and post (T2)-delivery to 6 weeks postpartum (T5). Multiple linear regression was utilised to compare TG and TEM between those with vaginal delivery (VD) and CS at each time point. Paired sample *t* -test with post hoc Bonferroni correction was utilised to compare laboratory markers over time.

**Results** Women in both groups had a median of three postpartum VTE risk factors, with higher body mass index and parity post-VD. In both the groups, TG and TEM parameters suggested hypercoagulability at T2 compared with T1, with resolution at T5. There were minimal differences between groups, apart from T2 with shorter clot formation time and higher maximum clot firmness in the VD group.

**Conclusion** TG and TEM illustrate hypercoagulability associated with pregnancy and delivery. The pattern of postpartum hypercoagulability seen in women with VTE risk factors was similar irrespective of mode of delivery. Further research is required to establish the effect of labour on TG/TEM in the absence of low molecular weight heparin use.

## Introduction

Venous thromboembolism (VTE) remains the leading cause of direct maternal mortality associated with pregnancy in the United Kingdom. 1 The incidence of pregnancy-associated VTE peaks in the postpartum period, with a fivefold increase in risk. 2 Identification of the at-risk woman and provision of thromboprophylaxis is key to reducing morbidity and mortality associated with VTE. The Royal College of Obstetricians and Gynaecologists (RCOG) recommends all pregnant women are risk assessed for VTE both antenatally at booking, during any hospitalisation and postdelivery. 3 4 The risk assessment incorporates both maternal risk factors (e.g. increased age or body mass index [BMI]) and those specific to pregnancy (e.g. twin pregnancy, emergency caesarean section [CS]). There is evidence to support mode of delivery as impacting on VTE risk; with elective CS associated with twofold increase in risk compared with vaginal delivery (VD) and further twofold increase in risk following emergency CS. 5 6 In England, the RCOG recommends all women following emergency CS, with BMI > 40 or with two VTE risk factors receive postpartum anticoagulant thromboprophylaxis for 7 days. 3 Those at very high risk including those with a personal history of VTE, high-risk thrombophilia or with three or more persistent risk factors should be offered extended postpartum thromboprophylaxis (anticoagulant and anti-embolism stockings [AES]) for 6 weeks.

Global coagulation assays such as thrombin generation and thromboelastometry (TEM) may provide a useful measure of hypercoagulability associated with pregnancy. TEM has demonstrated a hypercoagulable state in healthy pregnant women compared with age-matched controls 7 and with changes reflecting hypercoagulability increasing significantly in the second and third trimesters. 8 9 Similarly, thrombin generation has been demonstrated to increase from first to second trimester with a subsequent plateau in the third trimester. 10 11 12 13

In this study, we aimed to measure hypercoagulability in women requiring postpartum thromboprophylaxis at the time of delivery with thrombin generation and TEM, and to evaluate the impact of mode of delivery and thromboprophylaxis on these parameters. Specifically, we aimed to disprove or confirm the following null hypotheses:

1. There is no difference in thrombin generation between women with delivery by VD compared with CS in the peri- and postpartum period.
2. There are no differences in thrombin generation within the VD and CS groups over time (peri-/postpartum).
3. There is no difference in coagulability as measured by TEM between women delivering by VD versus by CS in the peri- and postpartum period.
4. There are no differences in TEM parameters within the VD and CS groups over time (peri-/postpartum).

## Materials and Methods

### Participants

Pregnant women were recruited from antenatal clinics where an indication for postnatal thromboprophylaxis was evident, from preassessment clinics prior to planned CS, and women admitted to the labour ward. The inclusion criteria were aged over 18 years with planned hospital delivery. Exclusion criteria were inability to provide informed consent, antenatal thromboprophylaxis or long-term anticoagulation, inability to return to follow-up, non-local residence, no indication for postpartum thromboprophylaxis or a contraindication to low molecular weight heparin (LMWH), AES or intermittent pneumatic compression devices (IPCDs). Data on patient demographics, VTE and bleeding risk factors, delivery and its complications were collected. The height and weight of all participants were measured to enable calculation of BMI.

Women were prescribed weight-based enoxaparin thromboprophylaxis (40 mg daily for weight 51–99 kg; 80 mg daily for weight 100–150 kg) on the basis of their postpartum VTE risk assessment (see Supplementary Material ) for 7 days or 6 weeks. Women delivering by CS had IPCD (Covidien, Hampshire, United Kingdom) applied while in theatre and until mobile on the ward, in addition to thigh length AES (Covidien). AES were provided to all women at high risk of VTE to continue for the same duration as enoxaparin, based on VTE risk assessment. Blood samples were collected at five time points: (T1/antepartum) on presentation in labour, at induction or prior to planned CS, (T2/postdelivery) after delivery, (T3/post-enoxaparin) at least 4 hours after first dose of enoxaparin, (T4/d7 postdelivery) at 7 days postdelivery, and (T5/6w postpartum) at 6 weeks postdelivery.

The study was approved by the London Riverside Research Ethics Committee (REC reference: 12/LO/1763) and King's College Hospital NHS Foundation Trust Research and Development department. All participants provided informed written consent prior to data and sample collection.

### Sample Collection

Blood was collected with 23G butterfly winged infusion sets into syringes (Greiner Bio-One, Kremsmünster, Austria), following an initial 5 mL discard draw, and then immediately dispensed into trisodium citrate vacuettes (0.109 mol/L) (VACUETTE, Greiner Bio-One). Corn trypsin inhibitor (CTI; Cambridge Biosciences, Cambridge, United Kingdom) was added to one vacuette prior to blood collection to give a final CTI concentration of 18.3 ug/mL. The first 5 mL of blood was divided into one K2E ethylenediaminetetraacetic acid vacuette (VACUETTE, Greiner Bio-One) and any other samples required as part of their routine clinical care.

### Laboratory Analysis

Plasma for international normalised ratio (INR), activated partial thromboplastin time (APTT), D-dimer, Clauss fibrinogen, anti-Xa activity and platelet-poor plasma (PPP) for thrombin generation was prepared as previously described. 14 In brief, PPP was prepared by double centrifugation (Hettich 46R Rotina Centrifuge, Tuttlingen, Germany) at 4,750 × *g* for 10 minutes, at room temperature. Following the first centrifugation, the top three-quarters of supernatant was decanted into a polypropylene tube and centrifuged a second time. The top three-quarter of the resulting supernatant was decanted into a plastic tube, frozen immediately and stored at –40°C within 60 minutes of sample collection. Standard coagulation assays were performed by senior biomedical scientists in the haematology laboratory as previously reported. 14 INR and APTT were measured by coagulation-based assays, with STA-Neoplastine and STA-Cephascreen, respectively. D-dimer was measured with a latex photometric immunoassay, using STA-Liatest. Fibrinogen was measured by the Clauss method, with STA-Fibrinogen. Anti-Xa activity was measured after thawing in a water bath at 37°C immediately prior to testing, with the STA-Rotachrom heparin colorimetric assay within 4 weeks of collection. Reagents were purchased from Diagnostica Stago (Asnieres, France) and assays were performed as per manufacturer's instructions on the fully automated STA-R Evolution analyser (Diagnostica Stago).

Thrombin generation was measured with calibrated automated thrombography (CAT) (Thrombinoscope BV, Maastricht, the Netherlands) as previously described in triplicate, 15 using the reconstituted PPP-LOW reagent to initiate thrombin generation (final concentration of 1 pM tissue factor and 4 μM phospholipids) (PPP-Reagent LOW, Thrombinoscope BV). Thrombin generation was measured in samples with and without CTI within 6 weeks of sample collection. The parameters measured were lag time, time to peak, peak thrombin and endogenous thrombin potential (ETP). Intra-assay variability was < 5% for all parameters and inter-assay variability was < 17% for all parameters.

TEM was performed on whole blood collected in 0.109 M trisodium citrated tubes following incubation at 37°C. All analyses were commenced within 30 minutes of sample collection. Reagents purchased from the manufacturer were used with the assay performed on the ROTEM Delta (Pentapharm GmbH, Münich, Germany) following manufacturer's instructions. Three assays were performed; INTEM, EXTEM and FIBTEM as previously described. 16 The parameters of interest were clotting time (CT), clot formation time (CFT) and maximum clot firmness (MCF).

### Statistical Analysis

Continuous variables are given as means and standard deviation or median and interquartile range (IQR) for normal and non-normal data, respectively. The means of continuous normally distributed variables were compared with the independent *t* -test between women with VD and CS with 95% confidence interval given for the difference in means. Non-normal data were compared between groups utilising independent the Mann–Whitney rank-sum test. Fisher's exact test was employed to compare nominal data between groups. A paired samples *t* -test was used to compare parameters within each group across different time points, with post hoc Bonferroni correction. To calculate the Bonferroni corrected *α* level, the original *α* -value (in this case 0.05) was divided by the number of analyses ( *α* / *n* ). For the comparison of CAT results by mode of delivery, an *α* -value of < 0.0025 was assigned, as this hypothesis involved 20 analyses. For the comparison of CAT variables between time points, an *α* -value of < 0.001 was applied, as the hypothesis involved a total of 80 tests (40 for VD and 40 for CS). For the comparison of TEM results by mode of delivery, an *α* -value of < 0.001 was assigned, as this hypothesis involved 35 analyses. For the comparison of CAT variables between time points, an *α* -value of < 0.001 was applied, as the hypothesis involved a total of 140 tests (70 for VD and 70 for CS). Predelivery (T1/antepartum) laboratory variables were compared with T2 (postdelivery) and T5 (6w postpartum) variables only. Comparisons were made between all postdelivery variables (T2–T5). Linear regression was undertaken to adjust for potential confounders (BMI, parity, active labour at T1, time from enoxaparin at T3) and for baseline coagulation parameters (6-week postpartum, T5 sample). Thrombin generation and TEM parameters were the dependent variable in all analyses. Further exploratory analyses were conducted to investigate the effect of labour and comparing VD to elective and emergency CS separately. Statistical significance was assumed at *p* < 0.05 for all exploratory analyses. All statistical analyses were undertaken on IBM SPSS version 21.0 software (SPSS, Chicago, Illinois, United States).

## Results

**Fig. 1:**
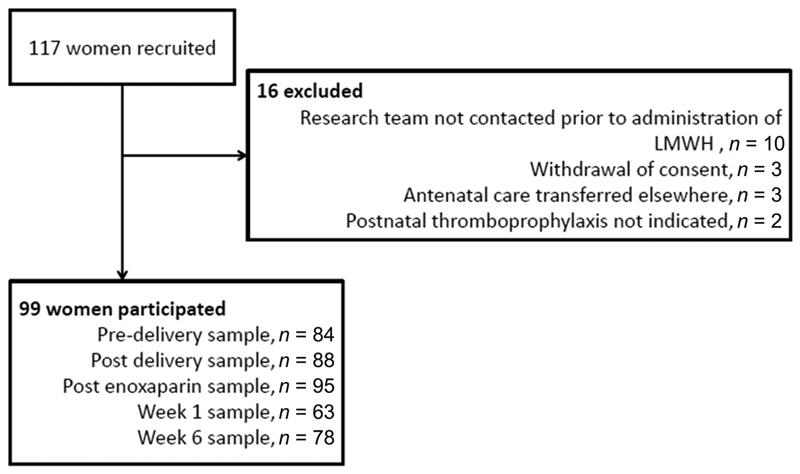
Study flow diagram.

One hundred and seventeen women were recruited; 99 provided blood samples both before and after enoxaparin administration (at least one of T1 or T2 and one of T3–T5) and were included in the study ( Fig. 1 ). There were 32 VD (including 7 instrumental deliveries) and 67 CS (42 elective, 25 emergency); at the time of emergency CS, 16 women were in active labour. Baseline characteristics are summarised in Table 1 . The proportion of women with two or more antenatal risk factors was higher in the VD group compared with CS (78.1% vs. 52.2%, *p* = 0.01); the proportion of women with two or more postnatal risk factors was not significantly different between groups (96.9% vs. 92.5%, respectively, *p* = 0.7). The median number of postpartum VTE risk factors in both groups was 3, with higher parity in the VD group (parity ≥ 2, 59.4% vs. 25.4% in the CS group, *p* = 0.001). No other significant differences in the prevalence of risk factors were seen. Of note, none of the women had known thrombophilia. All women were prescribed enoxaparin for at least 7 days, with the majority receiving 40 mg daily ( *n* = 76, 76.7%). Enoxaparin 80 mg daily was prescribed for 24 women (based on weight ≥ 100 kg). The median time to first dose of enoxaparin postdelivery was 11 hours (IQR, 8–16). Twenty-four (24.2%) women continued enoxaparin for 6 weeks (21 on 40 mg daily and 3 on 80 mg daily). There were no thrombotic events during the study period.

**Table 1:** Baseline characteristics

Characteristic	Whole cohort *n* = 99	Vaginal deliveries *n* = 32	Caesarean sections *n* = 67
Mean age (SD)	35.8 (5.0)	36.5 (5.4)	35.4 (4.8)
Ethnicity, *n* (%)			
White European	64 (64.6)	19 (59.3)	45 (70.3)
African-Caribbean	33 (33.3)	13 (19.4)	20 (29.8)
Other	2 (2.1)	0	2 (3.0)
Mean BMI, kg/m ^2^ (SD)	29 (7.3)	31.2 (8.1)	27.9 (6.8)
Current smoker, *n* (%)	3 (3.0)	1 (3.1)	2 (3.0)
Parity, predelivery, *n* (%)			
0	33 (33.3)	5 (15.6)	28 (41.7)
1	30 (30.3)	8 (25.0)	22 (32.8)
2	23 (23.2)	10 (31.3)	13 (19.4)
3	13 (13.1)	9 (28.1)	4 (6)
Other VTE risk factors			
Assisted conception, *n* (%)	11 (11.15)	3 (9.4)	8 (11.9)
Multiple pregnancies, *n* (%)	13 (13.1)	4 (12.5)	9 (13.4)
Pre-eclampsia, *n* (%)	35 (35.4)	11 (34.4)	24 (35.8)
Infection, *n* (%)	10 (10)	5 (15.6)	5 (7.5)
PPH, *n* (%)	8 (8)	2 (6.3)	6 (9)
Median postpartum risk factors, *n* (IQR)	3 (2–4)	3 (2–3)	3 (2–4)
Breastfeeding at T4, *n* (%)	75 (75.8)	28 (87.5)	47 (70.1)
Planned enoxaparin duration, *n* (%)			
7 d	75 (75.8)	20 (62.5)	55 (82.1)
6 wk	24 (24.2)	12 (37.5)	12 (17.9)

Abbreviations: BMI, body mass index; IQR, interquartile range; PPH, postpartum haemorrhage; SD, standard deviation; VTE, venous thromboembolism.

Samples were provided at T1 (antepartum) by 84 (84.8%) women, T2 (postdelivery) by 88 (88.9%), at T3 (post-enoxaparin) by 95 (96%), at T4 (d7 postdelivery) by 63 (63.6%) and by 78 (78.8%) women at T5 (6w postpartum). T2 (postdelivery) samples were collected at a median of 3 (IQR, 2–6) hours postdelivery, with T3 (post-enoxaparin) at a median of 24 (IQR, 20–27) hours postdelivery and 14 (IQR, 6–17) hours post-enoxaparin. T4 (7d postdelivery) was collected at a mean of 8 (±1) days postdelivery at a median of 29 (IQR, 15–25) hours post-enoxaparin. T5 (6w postpartum) samples were collected at a median of 46 (IQR, 42–53) days postdelivery. The collection time at T3 (post-enoxaparin) was significantly sooner after enoxaparin administration in the VD group compared with the CS group (9.9 hours, IQR, 4.4–14.1 vs. 15.2 hours, IQR, 9.8–17.6; *p* = 0.001). There were otherwise no significant differences in time of sample collection between groups (data not shown).

### Standard Coagulation Assays

INR and APTT were within normal limits at all time points with no significant differences detected between groups or time points (data not shown). D-dimer was significantly increased at T1 to T4 in both groups, with values peaking at T2 (postdelivery) and results within normal range at T5 (6w postpartum). Clauss fibrinogen was increased in both groups at T1 to T4 with results within reference range at T5 (6w postpartum). There were differences by mode of delivery over time as shown in Table 2 . Median anti-Xa activity at T3 (post-enoxaparin) and T4 (d7 postdelivery) were 0.07 IU/mL (IQR, 0.04–0.14) and 0.06 IU/mL (0.04–0.10), respectively.

**Table 2:** Comparison of median D-dimer and mean Clauss fibrinogen between women with vaginal delivery compared with caesarean section

	*N*	Median D-dimer, μg/mL (IQR)	Mean fibrinogen, g/dL (SD)
T1/antepartum	85		
Vaginal delivery		1780 (1010–2115)	5.4 (0.6)
Caesarean section		2140 (1402.5–2827.5)	4.9 (0.8)
Mean difference (95% CI)		–	−0.5 (−0.9, −0.1)
* p* -Value		0.063	0.007
T2/postdelivery	85		
Vaginal delivery		2730 (2190–3670)	5.0 (0.6)
Caesarean section		3859 (2705–6755)	4.3 (1.0)
Mean difference (95% CI)		–	−0.7 (−1.1, −0.3)
* p* -Value		0.006	0.001
T3/post-enoxaparin	95		
Vaginal delivery		1725 (1047–2297.5)	5.0 (0.7)
Caesarean section		2690 (1650–3940)	5.2 (0.8)
Mean difference (95% CI)		–	0.3 (−0.1, 0.6)
* p* -Value		< 0.001	0.12
T4/d7 postpartum	63		
Vaginal delivery		1060 (644–3182.5)	4.5 (0.7)
Caesarean section		2230 (1310–3720)	5.1 (0.9)
Mean difference (95% CI)		–	0.6 (0.2, 1.0)
* p* -Value		0.021	0.013
T5/6w postpartum	78		
Vaginal delivery		305 (270–480)	3.3 (0.9)
Caesarean section		350 (270–492.5)	3.5 (0.7)
Mean difference (95% CI)		–	0.1 (−0.2, 0.5)
* p* -Value		0.404	0.5

Abbreviations: CI, confidence interval; IQR, interquartile range; SD, standard deviation; -, not applicable.

### Thromboelastometry

**Fig. 2:**
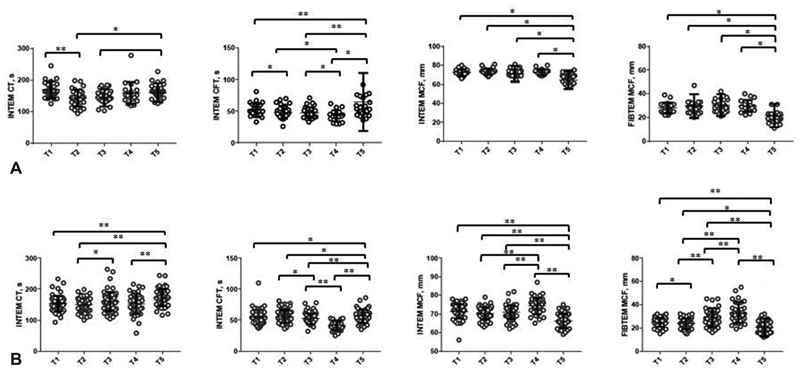
INTEM and FIBTEM parameters in women before (T1) and after vaginal delivery ( **A** ) or caesarean section ( **B** ) (T2), following enoxaparin (T3), at 1 week postpartum (T4) and 6 weeks postpartum (T5). * *p* < 0.001, ** *p* > 0.001 and < 0.05. Following a Bonferroni correction, significance was assumed at *α* < 0.001.

TEM profiles were hypercoagulable at T1 (antepartum) and T2 (postdelivery) with shortened CT, CFT and increased MCF on INTEM and FIBTEM MCF compared with T5, refer to Fig. 2 . These changes were attenuated following the first dose of enoxaparin (T3, post-enoxaparin). There were no significant differences in TEM parameters between VD and CS at T5 (6w postpartum). Similar changes were seen with EXTEM parameters (data not shown).

The differences in parameters by mode of delivery are summarised in Table 3 , and suggest hypercoagulability in the VD group at T2 (postdelivery), compared with CS. At 6 weeks postdelivery, INTEM MCF and FIBTEM MCF were significantly reduced in both groups compared with predelivery. Changes in TEM profiles throughout testing period are shown in Fig. 2 .

**Table 3:** Comparison of TEM parameters in vaginal deliveries compared with caesarean sections, showing the means ± 1 SD for each group

	EXTEM CT (s)	EXTEM CFT (s)	EXTEM MCF (mm)	INTEM CT (s)	INTEM CFT (s)	INTEM MCF (mm)	FIBTEM MCF (mm)
T1/antepartum							
Vaginal delivery	50.9 ± 6.1	58.1 ± 11.1	74.2 ± 3.1	168.8 ± 26.7	51.7 ± 10.6	72.3 ± 3.4	28.0 ± 4.4
Caesarean section	53.5 ± 6.7	59.5 ± 10.9	73.5 ± 3.4	153.1 ± 25.6	54.7 ± 11.8	71.3 ± 3.9	24.7 ± 4.4
Mean difference (95% CI)	2.6 (−0.6 to 5.6)	−1.4 (−3.7 to 6.7)	0.7 (−2.3 to 0.9)	15.7 (−28.1 to −3.2)	3 (−2.5 to 8.5)	1 (−2.7 to 0.9)	3.3 (−5.2 to −1.3)
* p* -Value	0.107	0.593	0.371	0.014 (0.071 adjusted)	0.284	0.312	0.002 (0.011 adjusted)
T2/postdelivery							
Vaginal delivery	49.5 ± 5.5	59.3 ± 21.9	72.7 ± 9.4	142.9 ± 26.4	48.3 ± 10	73.2 ± 3.1	28.0 ± 5.2
Caesarean section	50.3 ± 5.9	63.5 ± 12	72.3 ± 3.2	149.3 ± 21.3	55.9 ± 10.4	70.4 ± 3.4	23.5 ± 4.5
Mean difference (95% CI)	−0.8 (−1.8 to 3.4)	−4.2 (−3 to 11.4)	0.4 (−3.2 to 2.3)	−6.4 (−4.1 to 16.8)	−7.6 (3.0–10.2)	2.8 (−4.3 to 1.3)	4.5 (6.6−2.3)
* p* -Value	0.538	0.245	0.750	0.229	0.002 (0.002 adjusted)	0.001 (0.007 adjusted *)*	0.001 (0.001 adjusted)
T3/post-enoxaparin							
Vaginal delivery	51.2 ± 8.6	57.3 ± 13.3	74 ± 3.1	148.0 ± 20.3	46.8 ± 7.3	72.6 ± 3.6	28.8 ± 4.6
Caesarean section	51.7 ± 8.8	57.6 ± 12	73.4 ± 3.4	159.7 ± 30.6	52.1 ± 8.8	70.9 ± 3.7	28.3 ± 6.1
Mean difference (95% CI)	−0.5 (−3.3 to 4.2)	−0.3 (−5.1 to 5.7)	0.6 (−2.2 to 0.7)	−6.0 (−0.23 to 23.6)	−5.3 (−1.6 to −8.8)	1.6 (0.13–3.2)	0.47 (−2.9 to 2.0)
* p* -Value	0.809	0.908	0.329	0.054 (0.008 adjusted)	0.005 (0.02 adjusted *)*	0.033 (0.075 adjusted)	0.702
T4/d7 postdelivery							
Vaginal delivery	57.5 ± 5.9	48 ± 8.6	74.7 ± 2.8	159 ± 35.3	42.9 ± 8.7	72.6 ± 3.1	29.8 ± 4.9
Caesarean section	57.6 ± 11.4	45.6 ± 7.7	76.4 ± 3.4	152.2 ± 32.4	41.7 ± 7.3	74.2 ± 4.3	33.3 ± 8.9
Mean difference (95% CI)	−0.1 (−5.3 to 5.5)	2.3 (−6.7 to 2)	−1.7 (−0/01 to 3.5)	6.8 (−24.8 to 11.3)	1.2 (−5.4 to 3.0)	−1.6 (−0.5 to 3.8)	3.5 (−0.8 to 7.8)
* p* -Value	0.976	0.288	0.052	0.457	0.571	0.139	0.106
T5/6w postpartum							
Vaginal delivery	54.9 ± 6.8	66.8 ± 16.4	69.3 ± 4.2	162.7 ± 23.6	56.4 ± 13.3	66.3 ± 4.3	20.0 ± 4.3
Caesarean section	58.5 ± 7.2	63 ± 12.6	69.3 ± 3.7	172.8 ± 27.3	56.2 ± 10.7	66.3 ± 3.7	19.8 ± 4.9
Mean difference (95% CI)	−3.6 (0.3–7)	3.8 (−10.4 to 2.8)	0.1 (−1.7 to 1.9)	−6.1 (−2.1 to 22.3)	0.19 (−5.7 to 5.3)	−0.07 (−1.8 to 1.9)	−0.24 (−2.4 to 2.0)
* p* -Value	0.032 (0.032 adjusted)	0.253	0.922	0.105	0.945	0.940	0.829

Abbreviations: adjusted, adjusted for confounders; BMI, body mass index; CFT, clot formation time; CI, confidence interval; CT, clotting time; MCF, maximum clot firmness; SD, standard deviation; TEM, thromboelastometry.

Note: 95% confidence intervals of the difference between means and corresponding *p* -values (following adjustment in cases of significance for BMI, parity, active labour prior to sampling at T1, time since enoxaparin for T3 values and baseline values [as defined as T5]). Following a Bonferroni correction, significance was assumed at *α* < 0.001.

### Thrombin Generation

**Fig. 3:**
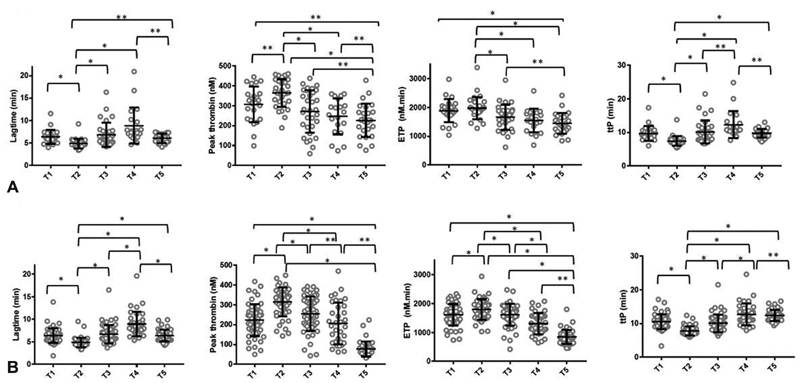
Thrombin generation parameters in women before (T1) and after vaginal delivery ( **A** ) or caesarean section ( **B** ) (T2), following enoxaparin (T3), at 1 week postpartum (T4) and 6 weeks postpartum (T5). * *p* < 0.001, ** *p* > 0.001 and < 0.05. Following a Bonferroni correction, significance was assumed at *α* < 0.001.

Changes in thrombin generation measured in the absence of CTI are shown in Fig. 3 . Thrombin generation profiles at T2 (postdelivery) were hypercoagulable with reduced lag time, time to peak and increased peak thrombin and ETP compared with T1 (antepartum). These changes were attenuated post first dose of enoxaparin (T3). At T5 (6w postpartum), lag time and time to peak were significantly longer compared with T2 (postdelivery) with reduced peak thrombin and ETP, suggesting a return to baseline levels. The pattern seen was similar in samples collected both with and without CTI (data not shown).

The differences in parameters by mode of delivery in non-CTI samples are summarised in Table 4 , with no significant differences between groups detected at any time point post-Bonferroni correction. Changes in thrombin generation parameters over time are illustrated in Fig. 3 .

**Table 4:** Comparison of CT parameters between women with vaginal delivery compared with those with caesarean section, showing the means =/– 1 SD for the values in each group

	Lag time (min)	ETP (nM/min)	Peak thrombin (nM)	Time to peak (min)
T1/antepartum				
Vaginal delivery	6.4 (1.5)	1887 (402.9)	306.7 (89.8)	9.7 (2.2)
Caesarean section	6.4 (1.6)	1608.6 (373)	222.7 (80.5)	10.6 (2.2)
Difference between groups (95% CI)	0 (−0.73 to 0.8)	278.4 (−464.7 to −92.2)	84 (124.8 to −43.6)	−0.9 (−0.17 to 1.9)
*p* -Value	0.90	0.005 (0.86 adj)	0.001 (0.17 adj)	0.10
T2/postdelivery				
Vaginal delivery	4.9 (1.1)	1972 (385)	363.6 (70.1)	7.4 (1.5)
Caesarean section	4.8 (1)	1794 (355.6)	314 (72)	7.8 (1.4)
Difference between groups (95% CI)	0.1 (−0.5 to 0.5)	178 (−347.4 to −8.6)	49.6 (−82.2 to −16.1)	−0.4(−0.3 to 0.99)
*p* -Value	0.90	0.04 (0.567 adj)	0.004 (0.897 adj)	0.30
T3/post-enoxaparin				
Vaginal delivery	6.8 (2.7)	1663.8 (425.9)	271.1 (105.8)	10.1 (3.4)
Caesarean section	6.7 (2)	1613.8 (378.7)	255.7 (87)	10.1 (2.6)
Difference between groups (95% CI)	0.1 (−1.2 to 0.9)	50 (−227.8 to 127.8)	15.4 (−57.3 to 26.5)	0 (−1.3 to 1.3)
*p* -Value	0.75	0.58	0.47	0.96
T4/d7 postdelivery				
Vaginal delivery	8.8 (4)	1544.7 (411)	247 (90.7)	12.3 (4)
Caesarean section	8.9 (2.7)	1357.7 (378)	206.3 (105)	12.7 (10.4)
Difference between groups (95% CI)	−0.1 (−1.7 to 1.9)	187 (−406.5 to 32.3)	40.7 (−97 to 15.6)	−0.4 (−1.6 to 2.4)
*p* -Value	0.93	0.09 (0.94 adj)	0.15	0.67
T5/6w postpartum				
Vaginal delivery	6.0 (1)	1443 (361.5)	226.6 (84)	9.7 (1.3)
Caesarean section	6.2 (1)	1192.7 (318.7)	156.3 (72.6)	10.6 (1.5)
Difference between groups (95% CI)	−0.2 (−0.4 to 0.6)	250.3 (−415.7 to −84.9)	70.3 (−108.4 to −32.4)	−0.9 (0.2–1.6)
*p* -Value	0.67	0.004 (0.042 adj)	< 0.001 (0.009 adj)	0.10

Abbreviations: BMI, body mass index; CI, confidence interval; CT, calibrated automated thrombography; ETP, endogenous thrombin potential; SD, standard deviation; T1-T5, time point 1–5.

Note: Confidence intervals of the mean difference and *p* -values reported for comparison of means using *t* -test, and following adjustment for BMI, active labour prior to sampling at T1, duration of labour and baseline values (as defined as T5). Following a Bonferroni correction, significance was assumed at *α* < 0.0025.

### Effect of Labour on Haemostatic Markers

At T1 (antepartum), 16 (VD = 12) women were in active labour at the time of blood sampling. These women exhibited significantly increased Clauss fibrinogen (5.4 ± 0.6 vs. 4.9 ± 0.8 g/L; *p* = 0.028), FIBTEM MCF (28 ± 5 vs. 25 ± 4 mm; *p* = 0.004), ETP (1950 ± 281 vs. 1628 ± 402 nM/min; *p* = 0.004) and peak thrombin (337 ± 64 vs. 225 ± 83 nM; *p* < 0.001) and shortened time to peak (9.1 ± 1.3 vs. 10.6 ± 2.3 minutes; *p* = 0.012) compared with women not in labour. At T2 (postdelivery), of the 88 women providing samples, 36 women had been in active labour predelivery (including 8 women with CS). Active labour prior to delivery was associated with significantly increased Clauss fibrinogen (5.0 ± 0.6 vs. 4.2 ± 1.0 g/L; *p* = 0.001), INTEM MCF (73 ± 3 vs. 70 ± 3 mm; *p* = 0.001), FIBTEM MCF (29 ± 9 vs. 23 ± 4 mm; *p* = 0.001), ETP (1975 ± 360 vs. 1756 ± 360 nM/min; *p* = 0.009) and peak thrombin (362 ± 66 vs. 206 ± 73 nM; *p* = 0.001) with significantly shorter INTEM CFT (49 ± 9 vs. 57 ± 11 seconds; *p* = 0.001) compared with women without prior active labour at T2 (postdelivery). No correlations were seen between duration of labour and haemostatic markers. At T3 (post-enoxaparin) and T4 (7d postpartum), there were no differences in any haemostatic markers between women with or without prior active labour.

Further exploratory sub-group analyses considering emergency and elective CS separately were attempted. Of note, the emergency CS numbers were small (T1 *n* = 18; T2 *n* = 14; T3 *n* = 18; T4 *n* = 12; T5 *n* = 13). Women with emergency CS had a significantly more hypercoagulable thrombin generation profile compared with women with elective CS at T1 (predelivery) only ( Supplementary Table S1 ), with no significant differences in thrombin generation at later time points (data not shown). Comparing elective CS to VD revealed the same pattern as the main analyses, with significant differences only at T5 (6w postpartum; data not shown). When the emergency CS group was compared with the VD group, there were no significant differences in thrombin generation between the groups at any time point, suggesting that active labour may play a part in the coagulation profile. The differences in Clauss fibrinogen seen in the main analysis were replicated on comparing VD with elective CS, but for VD compared with emergency CS, there were no significant differences in fibrinogen (data not shown). There were no significant differences in fibrinogen across time points between women with emergency CS compared with elective CS (data not shown). On subgroup analysis of D-dimer between VD with emergency and elective CS, there were similar findings to the main analysis ( Supplementary Table S2 ). Additionally, D-dimer was significantly higher in women with subsequent emergency CS compared with VD at T1 (antepartum). There were no significant differences in D-dimer between women with elective versus emergency CS. Differences in TEM parameters between VD, elective and emergency CS were similar to the main analysis ( Supplementary Tables S3 and S4 ). There were no significant differences in TEM parameters between elective and emergency CS (data not shown).

## Discussion

We investigated markers of hypercoagulability around the time of delivery in women qualifying for postpartum thromboprophylaxis. To our surprise, mode of delivery had little impact on thrombin generation or TEM parameters, with the key findings being evidence of hypercoagulability on TEM in women with VD immediately postdelivery. These women also exhibited higher peak and ETP at 6 weeks postpartum (T5) compared with those with CS (but without reaching significance). Further exploratory analyses separating emergency and elective CS, suggested a similar pattern of hypercoagulability to the main analysis but was limited by the small numbers of women undergoing emergency CS.

Additionally, we report the evolution of hypercoagulability in the peri-/postpartum period and the effect of enoxaparin; there was evidence of hypercoagulability immediately postdelivery with shortened lag time, time to peak and increased peak thrombin and ETP. These changes were attenuated following enoxaparin and continued to resolve at 1 week postdelivery. Similarly, TEM demonstrated hypercoagulability on the INTEM and FIBTEM assay in both groups immediately postdelivery with persistent changes at 1 week postpartum. At 6 weeks postpartum, hypercoagulability had resolved on both assays (compared with earlier time points) in keeping with prior reports. 9 12 17 18 19

In contrast, D-dimer was higher in women with CS following delivery until 6 weeks postpartum, with increased fibrinogen 1 week postpartum. Fibrinogen was increased in women with VD predelivery and immediately postdelivery compared with women with CS. These differences had resolved by 6 weeks. D-dimer has been previously reported to increase following delivery with subsequent return to normal levels by 4 weeks postpartum, and with more marked increase following CS compared with VD. 20 Fibrinogen has also been reported to increase following delivery with normalisation by day 19. 18 Previous disparity between D-dimer and thrombin generation has been reported in other settings, for example, following major orthopaedic surgery. 21 This is likely due in part to D-dimer reflecting prior in vivo clot formation, in contrast to thrombin generation which measures the ex vivo potential of plasma to generate thrombin. Increased thrombin–anti-thrombin levels have been reported post-CS compared with VD suggesting increased in vivo activation of coagulation and supporting this hypothesis. 22

### Strengths and Weaknesses

The strengths of our study include prospective recruitment of high VTE risk women, high rate of study completion (78.8%) and the use of global coagulation assays sensitive to the hypercoagulable state. 23 Thrombin generation has an established role as a research tool in demonstrating hypercoagulability with recognised independent associations with risk of both first and recurrent VTE 24 25 26 27 28 and sensitivity to hypercoagulable states including pregnancy, increasing age, ethnicity, obesity, heritable thrombophilia and hormone replacement therapy. 12 13 14 29 30 31

Limitations of our study include the lack of control group, small number of women in active labour at the time of CS, variable timing of sample collection postdelivery/administration of enoxaparin and incomplete sample provision at each time point. Given the standard of care in England is to provide thromboprophylaxis to women with two or more postpartum risk factors following VD (and this was a study inclusion criterion), it would have been unethical to include a ‘no treatment’ arm. However, it is possible that the use of enoxaparin in all participating women masked a later differentiation in hypercoagulability. We included women delivering by elective CS irrespective of other risk factors in keeping with our local VTE guidance to offer postpartum thromboprophylaxis for all CS; of note in our cohort 98.5% of women had at least one additional VTE risk factor. We previously reported 93.6% of women delivering by elective CS qualified for anticoagulant prophylaxis with RCOG risk stratification; supporting our approach to offer it to all such women. 32 We did not recruit a ‘low VTE risk’ group. While we attempted additional exploratory analyses of the effect of active labour on haemostatic markers, we were limited by the small number of women in labour at the time of sampling. Active labour was associated with both TEM and thrombin generation markers of hypercoagulability prepartum and immediately postpartum. The longer duration of labour for VD may contribute to hypercoagulability both directly (which was adjusted for in analyses) and potentially indirectly through factors such as dehydration (which were not measured/adjusted for). Thus, further study incorporating a low risk group would be valuable to evaluate whether the changes found are secondary to labour alone or moderated by the presence of VTE risk factors. Finally, it was not possible to standardise the sample collection time postdelivery/LMWH administration, due to variation in time of delivery. We therefore took a pragmatic approach to collecting when possible, and adjusted for timing when appropriate.

### Interpretation

TEM has been previously reported to demonstrate hypercoagulability in pregnancy. 33 Results from our cohort fall within previously proposed normal ranges for the peri-partum period but were skewed towards the proposed upper/lower limit of normality. 33 This is not unexpected given that we recruited women at high risk of VTE. TEM was insensitive to prophylactic enoxaparin; while INTEM CT was prolonged in the CS group at T3 (post-enoxaparin), there was no correlation between INTEM CT and anti-Xa activity (data not shown). A recent study utilising thromboelastography (TEG) to investigate the effect of prophylactic tinzaparin supports our findings; no detectable anticoagulant effect was seen at 4 to 10 hours following administration (with undetectable anti-Xa activity). 34 An in vitro study investigating the effect of dalteparin in whole blood collected from pregnant women revealed a dose-dependent effect on TEG only when anti-Xa activity was greater than 0.25 U/mL. 28 35

The changes in thrombin generation seen over time in our study compare with that previously reported in women receiving LMWH 12 19 with attenuation of hypercoagulability following first dose of enoxaparin (T3), and further at 1 week postdelivery. This may reflect physiological resolution of hypercoagulability in addition to anticoagulant effect. In contrast to our findings, Ismail et al reported higher ETP and peak thrombin in women with low VTE risk following VD compared with those following elective CS. 22 There was no significant change in thrombin generation pre- to post-CS and they did not measure predelivery thrombin generation in the VD group. It is therefore unclear whether the changes reported relate to mode of delivery, labour or other patient characteristics. It may be that global assays do not fully reflect underlying VTE risk given our findings do not support the well-established risk profile associated with mode of delivery 5 6 (highest with emergency CS and lowest with VD). This may be explained by additional factors not measured with these assays such as micro-trauma to pelvic vasculature, endothelial activation and consumption of coagulation factors intra-operatively. Measurement of thrombin–anti-thrombin levels may help elucidate this further.

Six-week postpartum (T5) thrombin generation parameters suggested hypercoagulability in women with VD compared with CS (but did not reach significance post-Bonferroni correction). This was not explained by ‘single factors’ but may result from synergy between multiple non-significant factors, with a greater proportion of women in this group having two or more VTE risk factors prior to delivery. The persistence of hypercoagulability at 1 week postpartum in our and former studies supports the role for extended thromboprophylaxis in women with very high VTE risk. 12 17 18 19 The optimal duration of thromboprophylaxis remains unknown with laboratory data suggesting 6 weeks may be excessive. However, clinical studies suggest the increased risk of VTE persists for up to 12 weeks postpartum. 36

## Conclusion

We have demonstrated that women with VTE risk factors post-VD have a similar pattern of postpartum hypercoagulability to women with CS. Further research is required to better define peri-partum hypercoagulability; specifically examining the effect of labour on haemostatic markers and TEM/thrombin generation profiles in the absence of LMWH use.
