# Supplementary material for: Mode of Delivery Does Not Influence Postpartum Hypercoagulability Measured by Thrombin Generation or Thromboelastometry
Source: TH Open. 2020 Jan 7;4(1):e1–e11. doi: 10.1055/s-0039-3402807 (PMC6946610; doi:10.1055/s-0039-3402807)
Supplement: Supplementary file 1 — Supplementary Material [file 10-1055-s-0039-3402807-s190052.pdf]

Supplementary Table S1 Comparison of thrombin generation by type of caesarean section at T1 (antepartum)

Time Point 1	*N*	Lag time (min)	ETP (nM/min)	Peak thrombin (nM)	Time to peak (min)
Elective CS mean (SD)	37	6.7 (1.7)	1527 (381.9)	209 (81)	11 (2.2)
Emergency CS mean (SD)	18	5.8 (1.3)	1776 (299.1)	251 (75)	9.6 (1.9)
Mean difference, 95% CI		0.9, 0.04–1.8	−249, −455 to 43	−42, −87 to 3.7	1.4, 0.2–2.7
p-Value		0.04	0.02	0.07	0.02

Abbreviations: CI, confidence interval; CS, caesarean section; ETP, endogenous thrombin potential; SD, standard deviation.

Supplementary Table S2 Comparison of D-dimer (ng/mL) across subgroups including elective and emergency caesarean section, showing median and IQR with *p*-values

	N	Vaginal delivery	*N*	Elective CS	*N*	Emergency CS	*p*-Value
T1	24	1780 (1010–2115)	40	1850 (1285–2965)	20	2441 (1552–2610)	0.03[b]
T2	29	3190 (2190–3670)	40	4491 (2557–6755)	16	4507 (3082–7247)	0.02[a]
T3	32	1798 (1048–3670)	41	3409 (1575–4695)	22	2700 (1920–3152)	0.001[a]
T4	20	1966 (644–3182)	28	2090 (1175–2905)	15	2250 (1540–8000)	0.012[b]
T5	28	423 (270–480)	33	320 (270–450)	17	819 (290–885)	NS

Abbreviations: CS, caesarean section; IQR, interquartile ranges; T1, predelivery; T2, postdelivery; T3, post-enoxaparin; T4, 1w postpartum; T5, 6w postpartum; VD, vaginal delivery.
[a]For both VD vs. emergency CS and VD vs. elective CS.
[b]For VD vs. emergency CS.

Supplementary Table S3 Comparison of TEM parameters in women with vaginal deliveries compared with elective caesarean sections, showing the means ± 1 SD for each group following adjustment in cases of significance for BMI, parity, time since enoxaparin for T3 and baseline values (defined as T5)

	EXTEM CT (s)	EXTEM CFT (s)	EXTEM MCF (mm)	INTEM CT (s)	INTEM CFT (s)	INTEM MCF (mm)	FIBTEM MCF (mm)
T1							
Vaginal delivery, *n* = 24	50.9 ± 6.1	58.1 ± 11.1	74.2 ± 3.1	168.8 ± 26.7	51.7 ± 10.6	72.3 ± 3.4	28.0 ± 4.4
Elective caesarean section, *n* = 39	53.1 ± 6	60 ± 12.2	73.3 ± 3.5	153.7 ± 24.2	55.8 ± 12.8	70.9 ± 4	24.8 ± 4
Mean difference (95% CIs)	2.2 (−1 to 5.3)	1.9 (−4.2 to 8)	−1.0 (−2.7 to 0.8)	−15.1 (−28.2 to −2)	4.1 (−2.1 to 10.4)	−1.4 (−3.4 to 0.6)	1.1 (−5.4 to −1)
p-Value	0.17	0.54	0.28	0.024 (0.16 adj)	0.19	0.17	**0.004 (0.008 adj)**
T2							
Vaginal delivery, *n* = 30	49.5 ± 5.5	59.3 ± 21.9	72.7 ± 9.4	142.9 ± 26.4	48.3 ± 10	73.2 ± 3.1	29.7 ± 10.1
Elective caesarean section, *n* = 41	49.5 ± 5.6	63.2 ± 12.4	72.4 ± 3.1	146.3 ± 17.8	56.1 ± 11.1	70.5 ± 3.4	23.3 ± 4.2
Mean difference (95% CIs)	−0.03 (−2.7 to 2.7)	3.9 (−4.2 to 12.1)	−0.3 (−3.5 to 2.8)	3.4 (−7.1 to 13.9)	−7.8 (2.7 to 12.9)	−2.7 (−4.3 to 1.1)	6.3 (−9.9 to −2.8)
p-Value	0.998	0.34	0.83	0.50	0.003 (0.081 adj)	**0.001 (0.006 adj)**	**0.001 (0.025 adj)**
T3							
Vaginal delivery, *n* = 32	51.2 ± 8.6	57.3 ± 13.3	74 ± 3.1	144.4 ± 27.3	47.7 ± 8.3	71.1 ± 8.3	30.2 ± 9.2
Elective caesarean section, *n* = 40	50.4 ± 9.2	58.9 ± 11.6	72.8 ± 3.2	151.9 ± 23	53 ± 9.3	70.3 ± 3.7	29.7 ± 8.5
Mean difference (95% CIs)	−0.9 (−5.1 to 3.4)	−1.6 (−4.2 to 7.5)	−1.3 (−2.8 to 0.2)	6.7 (−5.1 to 18.5)	5.3 (1.1–5.5)	−0.8 (−3.7 to 2.1)	1.5 (−5.7 to 2.7)
p-Value	0.68	0.58	0.09	0.26	**0.015 (0.035 adj)**	0.59	0.49
T4							
Vaginal delivery, *n* = 20	57.5 ± 5.9	48 ± 8.6	74.7 ± 2.8	159 ± 35.3	42.9 ± 8.7	72.6 ± 3.1	29.8 ± 4.9
Elective caesarean section, *n* = 28	54.7 ± 8.2	44.9 ± 7.6	76.4 ± 3.4	152.1 ± 35.3	42.9 ± 7.5	74 ± 4.4	33 ± 8
Mean difference (95% CIs)	−2.8 (−7.1 to 1.6)	3.1 (−7.8 to 1.6)	−1.7 (−0.1 to 3.6)	−6.9 (−27.7 to 13.9)	0.08 (−4.7 to 4.8)	1.4 (−0.9 to 3.7)	3.2 (−0.5 to 7)
p-Value	0.20	0.20	0.07	0.51	0.97	0.23	0.12
T5							
Vaginal delivery, *n* = 28	54.9 ± 6.8	66.8 ± 16.4	69.3 ± 4.2	160.1 ± 30.1	64.6 ± 45.8	64.6 ± 9.7	21.6 ± 9.7
Elective caesarean section, *n* = 33	58.3 ± 7.2	63.9 ± 12.7	69.1 ± 3.4	173.8 ± 28.7	57.5 ± 11.3	66.1 ± 3.5	20.8 ± 8.8
Mean difference (95% CIs)	3.4 (0.2–7.1)	−2.8 (−10.3 to 4.6)	−0.2 (−2.1 to 1.8)	13.7 (−1.4 to 28.8)	−7.1 (−23.5 to 9.4)	1.5 (−2.5 to 5.4)	−0.9 (−5.6 to 3.9)
p-Value	0.06	0.45	0.87	0.07	0.40	0.43	0.72

Abbreviations: adj, adjusted; BMI, body mass index; CFT, clot formation time; CI, confidence interval; CT, clotting time; MCF, maximum clot firmness; SD, standard deviation; T1, predelivery; T2, postdelivery; T3, post-enoxaparin; T4, 1w postpartum; T5, 6w postpartum; TEM, thromboelastometry.

Supplementary Table S4 Comparison of TEM parameters in women with vaginal deliveries compared with emergency caesarean sections, showing the means ± 1 SD for each group, following adjustment in cases of significance for BMI, parity, time since enoxaparin for T3 and baseline values (defined as T5)

	EXTEM CT (s)	EXTEM CFT (s)	EXTEM MCF (mm)	INTEM CT (s)	INTEM CFT (s)	INTEM MCF (mm)	FIBTEM MCF (mm)
T1							
Vaginal delivery, $n = 24$	50.9 ± 6.1	58.1 ± 11.1	74.2 ± 3.1	168.8 ± 26.7	51.7 ± 10.6	72.3 ± 3.4	28.0 ± 4.4
Emergency CS, $n = 20$	54.4 ± 8.1	58.7 ± 8.2	74 ± 3.1	151.9 ± 28.9	52.5 ± 9.8	72.3 ± 3.5	24.4 ± 4
Mean difference (95% CIs)	3.4 (−0.9 to 7.8)	0.6 (−5.5 to 6.6)	−0.3(−2.2 to 1.6)	−16.9 (−33.8 to −0.04)	0.8 (−5.4 to 6.9)	0 (−2.1 to 2.1)	−3.3 (−5.8 to −0.7)
p-Value	0.12	0.85	0.78	0.051	0.80	1.0	**0.014 (0.035 adj)**
T2							
Vaginal delivery, $n = 30$	49.5 ± 5.5	59.3 ± 21.9	72.7 ± 9.4	142.9 ± 26.4	48.3 ± 10	73.2 ± 3.1	29.7 ± 10.1
Emergency CS, $n = 16$	52.4 ± 6.1	64.4 ± 11.3	72 ± 3.5	157.3 ± 27.8	55.3 ± 8.4	70.2 ± 3.3	24 ± 5.5
Mean difference (95% CIs)	2.8 (−0.7 to 6.4)	5.1 (−6.7 to 16.9)	−0.7 (−5.6 to 4.2)	14.4 (−2.7 to 31.6)	7 (0.9–13.1)	−3 (−5 to 1)	−5.7 (−11.4 to −0.01)
p-Value	0.12	0.39	0.78	0.10	**0.025 (0.23 adj)**	**0.004 (0.023 adj)**	**0.05**
T3							
Vaginal delivery, $n = 32$	51.2 ± 8.6	57.3 ± 13.3	74 ± 3.1	144.4 ± 27.3	47.7 ± 8.3	71.1 ± 8.3	30.2 ± 9.2
Emergency CS, $n = 22$	54.1 ± 7.4	55.2 ± 7.6	74.4 ± 3.6	175.7 ± 36.4	50.7 ± 8.2	71.9 ± 3.6	29.7 ± 6.9
Mean difference (95% CIs)	2.9 (−1.7 to 7.4)	−2.1 (−9.3 to 5.2)	0.3 (−1.5 to 2.2)	31.3 (13.9–48.7)	3 (−1.6 to 7.6)	−0.8 (−3 to 4.6)	−0.5(−5.2 to 4.2)
p-Value	0.21	0.57	0.71	**0.001 (0.001 adj)**	0.20	0.68	0.83
T4							
Vaginal delivery, $n = 20$	57.5 ± 5.9	48 ± 8.6	74.7 ± 2.8	159 ± 35.3	42.9 ± 8.7	72.6 ± 3.1	29.8 ± 4.9
Emergency CS, $n = 15$	62.9 ± 14.4	47.2 ± 8	76.5 ± 3.6	152.5 ± 27.4	39.3 ± 6.5	74.6 ± 4.2	33.8 ± 10.7
Mean difference (95% CIs)	5.4 (1.8–12.7)	−0.9 (−6.7 to 4.9)	1.8 (−0.4 to 4)	−6.5 (−28.8 to 15.9)	−3.6 (−9 to 1.9)	4.1 (−1.4 to 9.5)	3.2 (−0.5 to 7)
p-Value	0.14	0.76	0.11	0.56	0.19	0.14	0.12
T5							
Vaginal delivery, $n = 28$	54.9 ± 6.8	66.8 ± 16.4	69.3 ± 4.2	160.1 ± 30.1	64.6 ± 45.8	64.6 ± 9.7	21.6 ± 9.7
Emergency CS, $n = 18$	58.9 ± 7.4	61.1 ± 12.6	69.8 ± 4.3	170.7 ± 25	53.6 ± 9.2	66.8 ± 4.3	20.2 ± 4.8
Mean difference (95% CIs)	4 (0.3–8.3)	−5.7 (−15.1 to 3.6)	0.6 (−2 to 3.3)	10.6 (−7 to 28.1)	−10.9 (−33.7 to 11.8)	2.2 (−2.8 to 7.3)	−1.4 (−6.5 to 3.7)
p-Value	0.07	0.22	0.66	0.23	0.34	0.38	0.58

Abbreviations: adj, adjusted; BMI, body mass index; CFT, clot formation time; CI, confidence interval; CS, caesarean section; CT, clotting time; MCF, maximum clot firmness; SD, standard deviation; T1, predelivery; T2, postdelivery; T3, post-enoxaparin; T4, 1w postpartum; T5, 6w postpartum; TEM, thromboelastometry.
